# Determinants of HPV-vaccination uptake and subgroups with a lower uptake in the Netherlands

**DOI:** 10.1186/s12889-021-11897-0

**Published:** 2021-10-12

**Authors:** A. C. de Munter, T. M. Schurink-van t Klooster, A. van Lier, R. Akkermans, H. E. de Melker, W. L. M. Ruijs

**Affiliations:** 1 Department of Infectious Disease Control, Public Health Service Gelderland-Zuid, Nijmegen, The Netherlands; 2 Radboud University Nijmegen Medical Centre, Department of Primary and Community Care & IQ Health care, Nijmegen, The Netherlands; 3 Radboud University Medical Centre, Radboud Institute for Health Sciences, IQ healthcare, Nijmegen, The Netherlands; 4 Department National Immunization Program, Center of Epidemiology and Surveillance of Infectious Diseases, National Institute for Public Health and the Environment, PO box 1, 3720 BA Bilthoven, The Netherlands; 5 GGD GHOR Nederland, Utrecht, The Netherlands

**Keywords:** Immunization, Human papillomavirus (HPV), Ethnicity, Urbanization, Socioeconomic status, Political preference

## Abstract

**Background:**

In the Netherlands, the HPV-vaccine uptake was 52% during the 2009 catch-up campaign (birth cohorts 1993–1996). This increased to 61% in the regular immunization program (birth cohorts 2000–2001). However for birth cohorts 2003–2004 the uptake declined to 45.5%. With this study we aimed to gain insight into social, economic and cultural determinants that are associated with HPV-vaccination uptake and which subgroups with a lower HPV-vaccination uptake can be identified. In addition, we investigated whether the influence of these factors changed over time.

**Methods:**

To study the determinants of HPV-vaccine uptake we performed a database study using different aggregation levels, i.e. individual level, postal code level and municipality level. All Dutch girls who were invited for HPV-vaccination through the National Immunization Program in the years 2012, 2014 and 2017 (i.e. birth cohorts 1999, 2001 and 2004, respectively) were included in the study population. We conducted multilevel logistic regression analyses to analyze the influence of the determinants on HPV-vaccination uptake, taking into account that the delivery of HPV-vaccine was nested within municipalities.

**Results:**

Results showed that in particular having not received a MMR-vaccination, having one or two parents born in Morocco or Turkey, living in an area with lower socioeconomic status and higher municipal voting proportions for Christian political parties or populist parties with liberal-conservative views were associated with a lower HPV-vaccination uptake. Besides some changes in political preferences of the population and changes in the association between HPV uptake and urbanization level we found no clear determinants which could possibly explain the decrease in the HPV-vaccination uptake.

**Conclusions:**

In this study we identified current social, economic and cultural determinants that are associated with HPV-vaccination uptake and which low-vaccination subgroups can be identified. However, no clear determinants were found which could explain the decrease in the HPV-vaccination uptake. Tailored information and/or consultation for groups that are associated with a lower HPV-vaccination uptake might help to increase the HPV-vaccination uptake in the future.

**Supplementary Information:**

The online version contains supplementary material available at 10.1186/s12889-021-11897-0.

## Background

Vaccination against human papillomavirus (HPV) targeting girls 12 years of age is part of the Dutch national immunization program (NIP) since 2010. Prior to this, a catch-up campaign for 13–16-year-old girls was initiated in 2009. The bivalent HPV16/18-vaccine was used starting with a three-dose schedule up to 2013 and a two-dose schedule from 2014 onwards. HPV16 and − 18 together are estimated to account for 70% of all cases of cervical cancer [1]. In the Netherlands, annually about 800 women are diagnosed with cervical cancer and about 200 die due to this disease [2, 3].

The HPV-vaccination uptake is low compared to the coverage for other vaccines in the Dutch NIP. During the catch-up campaign in 2009, the vaccine coverage was 52% for birth cohorts 1993–1996 [4]. This increased to 61% for birth cohorts 2000 and 2001 but declined thereafter to 45.5% for birth cohorts 2003 and 2004 [5]. In addition, large variations in the vaccination coverage were observed at municipality level ranging from less than 10% to more than 80% [6].

Research among girls who were targeted for the initial catch-up campaign and their mothers showed that socio-demographic determinants, such as socioeconomic status (SES) and country of birth were associated with HPV-vaccination uptake [7, 8]. In addition, various Christian groups have objections to HPV-vaccination because it concerns protection against a sexually transmitted infection or because they have religious objections to vaccination in general [7, 9, 10]. Previous studies indicate that in several high income countries lack of trust in the government also plays a role in the willingness to get HPV-vaccination [11–13]. An ecological study conducted in the United States showed that political color is associated with vaccination uptake in adolescence, as well [14]. In the Netherlands, high political preference for Protestant-Christian parties at municipality level was previously found to be associated with low HPV-vaccination uptake [7]. Political preference for other political parties might also be associated with low HPV-vaccination uptake, because of the relation with confidence in government institutions, media and social institutions [15, 16].

It is unknown whether the influence of the various social, economic and cultural determinants on HPV-vaccination uptake changed over time in the Netherlands. In addition, it is unknown which determinants could explain the recent decrease in the HPV-vaccination uptake. With this study, we aim to gain insight into the determinants that are associated with HPV-vaccination uptake and which low-vaccination subgroups can be identified, and to investigate whether target groups can be identified that are associated with the decline in HPV-vaccination uptake.

## Methods

### Sample and data collection

We performed a database study to investigate various determinants of HPV-vaccination uptake on different aggregation levels: individual, postal code and municipality. The sample included all girls invited for HPV-vaccination through the NIP in the years 2012, 2014 and 2017, respectively from birth cohorts 1999, 2001 and 2004. For 2017 was the latest complete dataset available; in 2014 the vaccination schedule was changed and this was the last year before the decline in vaccination uptake; in 2012 and 2017 the Dutch National Elections for seats in the House of Representatives were held.

Anonymous individual-level data were obtained retrospectively in 2018 from the national vaccination register (Praeventis), using the 2018 municipality division (380 municipalities). The individual level variable Ethnicity was defined as country of birth of both parents, for which most common country of birth combinations were used.

Additional data, on postal code and municipality level, were extracted from the publicly available data of Statistics Netherlands (CBS), The Netherlands Institute for Social Research (SCP), and the Electoral Council (Kiesraad), or were provided by the Municipal Health Services (MHS). If data was not available for a certain invitation year, data of the most recent year was used (see Table 1 for variable details).

**Table 1:** Characteristics of variables: level of aggregation, measurement level, year of data collection for each invitation year and original database

Variable	Measurement level	Invitation Year^**1**^	Year of data collection^**2**^	Database
***Individual-level***				
HPV-vaccination status (dependent variable)	Dichotomous: Completed series of HPV-vaccinations; 0 = has no completed HPV-vaccination series; 1 = has a completed HPV-vaccination series (2012: 3-doses; 2014/2017: 2-doses)	2012 2014 2017	2018 2018 2018	Praeventis
MMR-vaccination status	Categorical: Zero, one, two doses of MMR-vaccination	2012 2014 2017	2018 2018 2018	Praeventis
DT (aP)-IPV-vaccination status	Categorical: Zero, primary series (3-doses), completed series (6-doses) of DT (aP)-IPV-vaccination	2012 2014 2017	2018 2018 2018	Praeventis
Ethnicity ^3^	Categorical: 14 combinations of parents’ country of birth^4^ and the category unknown (one or both parents’ country of birth is unknown)^5^	2012 2014 2017	2018 2018 2018	Praeventis
***Postal code-level***				
Socioeconomic status (SES)	Categorical: Status score low (≤ − 1.0000), low-intermediate (−0.9999 to 0.0000), high-intermediate (0.0001–0.9999), high (≥1.0000)	2012 2014 2017	2010 2014 2016	SCP
Road distance	Categorical: 0 km (HPV-vaccination provided in same postal code as home address), 0–5 (0.1–4.9) km, 5–10 (5.0–9.9) km, ≥10 km	2012 2014 2017	2014 2014 2017	MHS
***Municipality-level***				
Urbanization level^5^	Categorical: Very high (> 2500 addresses per km^2^), High (1500–2500 add. Per km^2^), Moderately high (1000–1500 add. Per km^2^), Low (500–1000 add. Per km^2^), Very low (< 500 add. Per km^2^)	2012 2014 2017	2017 2017 2017	CBS
Voting proportions from the National Elections for political parties^6^	Dichotomous: Voting proportion (percentage of votes per political party) lower or higher than the mean of the national voting proportion of the party.	2012 2014 2017	2012 2012 2017	Electoral Council

*Abbreviations: HPV* Human Papillomavirus, *MMR* Mumps-measles-rubella, *DTaP-IPV* diphtheria-tetanus-pertussis-polio, *SCP* The Netherlands Institute for Social Research, *MHS* Municipal Health Services, *CBS* Statistics Netherlands, *km* kilometer. *Praeventis* National vaccination registry

^1^ Girls invited for HPV-vaccination through the NIP in the years 2012, 2014 and 2017 were born in 1999, 2001 and 2004 respectively

^2^ If data was not available for a certain invitation year, data of the most recent year was used

^3^ From December 2002 onwards, parents’ country of birth was authorized from the Personal Records Database (Dutch: BRP, previously known as GBA) and therefore more complete for girls invited in 2017 (birth cohort 2004) than for girls invited in 2012 and 2014 (birth cohorts 1999 and 2001)

^4^ The Netherlands-The Netherlands, The Netherlands-Turkey, Turkey-Turkey, The Netherlands-Morocco, Morocco-Morocco, The Netherlands-Surinam, Surinam-Surinam, The Netherlands-Netherlands Antilles and Aruba, Netherlands Antilles and Aruba-Netherlands Antilles and Aruba, The Netherlands-other western country, other western country-other western country, The Netherlands-other non-western country, other non-western country -other non-western country, other western country-other non-western country, unknown

^5^ In the database the urbanization level of 2017 was used; the most recent HPV-vaccination invitation year. Following the municipal re-division between 2017 and 2018, several municipalities merged into three new municipalities. For these three new municipalities we used the urbanization level of 2018

^6^ Ten variables: 1) People’s Party for Freedom and Democracy (VVD; right-wing liberal party with more progressive positions in ethical matters), 2) Labour Party (PvdA; progressive, social-democratic party) & Denk (DENK; movement for migrants and a “tolerant and solidary society”; political party founded in 2015 by former members of the PvdA), 3) Party for Freedom (PVV; populist party with both conservative, liberal “right” and “left” views) & Forum for Democracy (FvD; conservative, right-wing populist Eurosceptic political party; political party founded in 2015, whose voters are mainly former PVV

voters), 4) Socialist Party (SP; socialist, Eurosceptic party which has a strong local, action-oriented basis), 5) Christian Democratic Appeal (CDA; Christian-inspired party at the center of the political spectrum), 6) Democrats 66 (D66; reformist social-liberal party), 7) Christian Union (CU; Christian party, with progressive positions in the social and ecological field and conservative positions on ethical issues) & Reformed Political Party (SGP; conservative Christian (Reformed) party that wants to conduct politics strictly according to Biblical standards), 8) Green Left (GL; progressive party which attaches great importance to sustainability), 9) Party for the Animals (PvdD; testimonial party with main goals animal rights and animal welfare), 10) 50PLUS (50+; party that stands up especially for the interests of people aged 50 and over). The voting proportions for the three new municipalities in 2018 were calculated based on the weighted averages of the voting proportions of the previous municipalities before they were merged into the new municipality.

The postal code level variable Socioeconomic status was defined as status score, which is calculated by the SCP based on the educational level, paid jobs and income of households. Road distance was defined as distance by car between girls’ home address and vaccination location in kilometers.

Voting proportions from the National Elections for political parties with 2 or more seats in the House were included in the analyses. Supplementary material 1 contains a list of these political parties and the distribution of seats in the House of Representatives in the Dutch National elections of 2012 and 2017 [17].

### Statistical analysis

**Fig. 1:**
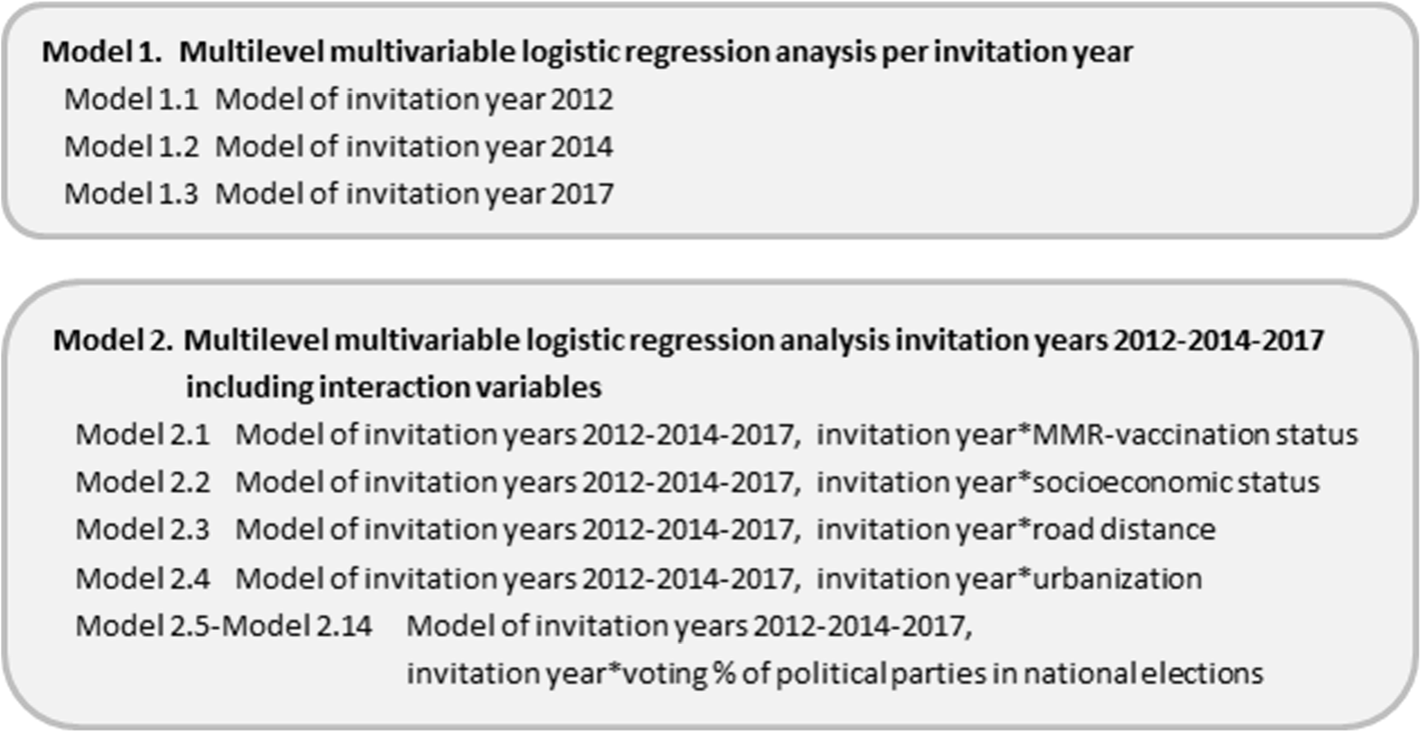
Multilevel multivariable models used for statistical analysis

Multilevel logistic regression analyses were used to determine the association between the dependent variable HPV-vaccination uptake of a completed series (2 or 3 doses depending on invitation year) and predictor variables. The multilevel models included two hierarchical levels where girls who were invited for HPV-vaccinations (level 1) were nested in municipalities (level 2). First, the associations between HPV-vaccination uptake and independent variables (Table 1) were measured using multilevel univariate logistic regression analyses [18]. Road distance to the vaccination location, SES and voting proportions for political parties were included on a categorical scale -instead of interval scale- to assess the relative effect of the predictor variables [19]. Secondly, multilevel multivariable logistic regression analysis was conducted. Predictor variables were selected based on a statistically significant association with HPV-vaccination uptake following the multilevel univariate logistic regression analysis (*p* < 0.05) unless multicollinearity (> 0.70) was found between two or more predictor variables. To calculate the correlation between all predictor variables in order to detect multicollinearity Spearman’s correlation coefficient and the phi coefficient (2 × 2) were used [18]. In the multilevel multivariable logistic regression analysis, we used two different main models (Fig. 1). Model 1 contained a separate multilevel multivariable logistic regression model for each of the invitation years (2012, 2014 and 2017). In model 2, we combined the data of three invitation years using an additional variable for invitation year (categorial) and an interaction variable invitation year*predictor variable, to measure the effect of change of the predictor variables over time.

All analyses were performed using IBM SPSS Statistics®, version 24. Associations between HPV-vaccination uptake and predictor variables are shown with crude odds ratios (COR), adjusted odds ratios (AOR) and 95% confidence intervals (95%CI).

### Ethical considerations

The study was approved by the research ethics committee of the Radboud University Nijmegen Medical Centre, Nijmegen, the Netherlands; CMO number 2018/4744.

## Results

In the following paragraphs, main results of the multilevel univariate and multivariable logistic analysis of the invitation year 2017 are presented per predictor variable. Additionally, these results are compared to the associations between HPV-vaccination uptake and the predictor variables in the invitation years 2012 and 2014. Tables of the multilevel univariate analysis (Table 2) and multilevel multivariable logistic regression model (Table 3; model 1.3 for girls invited for HPV-vaccination in 2017) are included in this article. Model 1.1. and 1.2. (for the girls invited in 2012 and 2014) and the models including the interaction variable between invitation years and each predictor variable (models 2.1–2.14) can be found in supplementary material 2.

**Table 2:** Descriptive analysis HPV-vaccination uptake and predictor variables on individual, postal code and municipality level (*n* = 299,883)

	2012				2014				2017			
Variable	N	HPV-uptake % ^(a)^	Multi- level univariate COR	***p***-value	N	HPV-uptake % ^(a)^	Multi- level univariate COR	p-value	N	HPV-uptake % ^(a)^	Multi- level univariate COR	p-value
**Total**	102,456	60.0%			100,988	62.8%			96,439	46.6%		
***Individual level***												
**MMR-vaccination status**	**2012**			**< 0.001**	**2014**			**< 0.001**	**2017**			**< 0.001**
Zero vaccinations	5355	3.7%	reference	ref.	3190	6.6%	ref.	ref.	2548	6.0%	ref.	ref.
One vaccination	3079	28.4%	9.72	< 0.001	3304	26.2%	4.68	< 0.001	3162	13.3%	2.24	< 0.001
Two Vaccinations	94,022	64.3%	44.05	< 0.001	94,494	66.0%	25.34	< 0.001	90,729	48.9%	13.95	< 0.001
**DT (aP)-IPV-vaccination status**	**2012**			**< 0.001**	**2014**			**< 0.001**	**2017**			**< 0.001**
Zero vaccinations	5549	6.1%	ref.	ref.	3258	10.3%	ref.	ref.	2656	8.2%	ref.	ref.
Primary series	2851	28.2%	5.67	< 0.001	3088	25.9%	2.81	< 0.001	3539	20.0%	2.63	< 0.001
Completed series	94,056	64.2%	25.84	< 0.001	94,642	65.8%	15.50	< 0.001	90,244	48.7%	9.94	< 0.001
**Ethnicity**	**2012**			**< 0.001**	**2014**			**< 0.001**	**2017**			**< 0.001**
NLD - NLD	17,319	63.6%	ref.	ref.	17,761	65.2%	ref.	ref.	70,614	49.4%	ref.	ref.
NLD - Turkey	352	32.4%	0.30	< 0.001	405	34.6%	0.30	< 0.001	1023	26.0%	0.34	< 0.001
Turkey - Turkey	1306	27.9%	0.26	< 0.001	1133	30.3%	0.25	< 0.001	1802	20.5%	0.24	< 0.001
NLD - Morocco	199	36.2%	0.36	< 0.001	224	35.7%	0.34	< 0.001	762	18.8%	0.22	< 0.001
Morocco - Morocco	1272	18.2%	0.16	< 0.001	1249	23.9%	0.20	< 0.001	2920	16.5%	0.18	< 0.001
NLD - Surinam	369	52.8%	0.73	0.003	414	54.8%	0.70	0.001	927	41.3%	0.71	< 0.001
Surinam - Surinam	544	48.5%	0.68	< 0.001	463	57.9%	0.86	0.108	708	45.6%	0.86	0.048
NLD - Ned Antilles/Aruba	186	51.6%	0.65	0.004	161	57.8%	0.74	0.058	475	35.6%	0.55	< 0.001
Ned Antilles/Aruba –Ned Antilles/Aruba	214	23.8%	0.20	< 0.001	150	44.7%	0.45	< 0.001	183	35.5%	0.57	< 0.001
NLD - other WC	748	58.6%	0.84	0.020	858	64.1%	0.95	0.493	2331	51.0%	1.03	0.573
other WC - other WC	430	43.5%	0.44	< 0.001	468	53.2%	0.62	< 0.001	704	46.9%	0.84	0.025
NLD - other NWC	490	58.0%	0.84	0.071	581	59.9%	0.82	0.027	1673	50.0%	0.99	0.778
other NWC - other NWC	1184	47.6%	0.57	< 0.001	1192	59.3%	0.83	0.004	2190	51.1%	1.05	0.252
other WC - other NWC	92	43.5%	0.50	0.001	104	44.2%	0.45	< 0.001	196	35.7%	0.53	< 0.001
Unknown	77,751	61.2%	0.85	< 0.001	75,825	63.9%	0.94	0.003	9931	42.9%	0.74	< 0.001
***Postal code level***												
**Socioeconomic status** ^(b)^	**2012**			**< 0.001**	**2014**			**< 0.001**	2017*			**< 0.001**
Low	17,723	51.4%	ref.	ref.	19,395	54.9%	ref.	ref.	18,149	36.9%	ref.	ref.
Low-intermediate	29,714	58.8%	1.27	< 0.001	26,556	62.9%	1.34	< 0.001	25,467	44.6%	1.36	< 0.001
High-intermediate	38,297	62.7%	1.50	< 0.001	37,747	64.4%	1.57	< 0.001	34,292	48.8%	1.64	< 0.001
High	15,981	65.3%	1.84	< 0.001	16,732	68.4%	1.93	< 0.001	18,099	54.8%	2.09	< 0.001
**Road distance**	2012*			**0.129**	**2014**			**0.031**	**2017**			**0.340**
0 km	12,694	60.3%	ref.	ref.	13,661	62.3%	ref.	ref.	13,104	45.8%	ref.	ref.
0–5 km	44,246	59.3%	1.03	0.279	41,395	62.0%	1.04	0.105	38,045	46.9%	1.02	0.547
5–10 km	28,986	60.6%	1.06	0.026	28,560	63.9%	1.07	0.003	29,079	46.7%	1.04	0.081
> 10 km	16,495	60.8%	1.05	0.115	17,343	63.3%	1.05	0.072	16,185	46.2%	1.02	0.333
***Municipality level***												
**Urbanization level** ^(c)^	2012*			**0.020**	2014*			**0.134**	**2017**			**0.058**
Very high	23,398	53.3%	ref.	ref.	19,446	57.9%	ref.	ref.	19,280	45.3%	ref.	ref.
High	31,870	60.3%	1.14	0.272	30,512	62.9%	1.03	0.793	29,502	46.1%	0.95	0.642
Moderately high	17,260	62.7%	1.28	0.038	18,314	64.0%	1.16	0.232	17,512	47.9%	1.03	0.819
Low	21,315	63.8%	1.36	0.007	23,200	65.5%	1.22	0.099	21,526	48.3%	1.07	0.572
Very low	8604	63.1%	1.25	0.062	9513	64.1%	1.11	0.390	8618	44.3%	0.87	0.266
**Voting % political parties** ^**(d)**^	**2012**				2014*				**2017**			
Lower or higher than national mean												
**People’s Party for Freedom and Democracy (VVD)**												
Lower	64,595	57.3%	ref.	ref.	60,833	60.5%	ref.	ref.	58,855	43.6%	ref.	ref.
Higher	37,852	64.8%	1.30	< 0.001	40,152	66.3%	1.27	< 0.001	37,583	51.3%	1.47	< 0.001
**Labor Party (PvdA), Denk (DENK)**												
Lower	36,543	60.9%	reference	ref.	39,038	62.7%	ref.	ref.	34,880	46.7%	ref.	ref.
Higher	65,904	59.6%	1.09	0.096	61,947	62.9%	1.10	0.076	61,558	46.5%	1.00	0.931
**Party for Freedom (PPV), Forum for Democracy (FvD)**												
Lower	57,814	59.7%	reference	ref.	56,784	62.3%	ref.	ref.	52,307	48.1%	ref.	ref.
Higher	44,633	60.5%	1.14	0.007	44,201	63.5%	1.10	0.077	44,131	44.8%	0.88	0.006
**Socialist Party (SP)**												
Lower	52,335	57.6%	reference	ref.	53,199	59.8%	ref.	ref.	4665	44.3%	ref.	ref.
Higher	50,112	62.6%	1.40	< 0.001	47,786	66.2%	1.45	< 0.001	41,773	49.5%	1.29	< 0.001
**Christian Democratic Appeal (CDA)**												
Lower	77,344	59.9%	ref.	ref.	73,747	63.0%	ref.	ref.	71,075	46.8%	ref.	ref.
Higher	25,103	60.6%	0.94	0.201	27,238	62.4%	0.97	0.487	25,363	45.9%	0.94	0.221
**Democrats 66 (D66)**												
Lower	43,514	59.9%	ref.	ref.	45,849	61.2%	ref.	ref.	38,093	42.7%	ref.	ref.
Higher	58,933	60.1%	1.25	< 0.001	55,136	64.2%	1.30	< 0.001	58,345	49.1%	1.46	< 0.001
**Christian Union (CU), Reformed Political Party (SGP)**												
Lower	78,482	61.8%	ref.	ref.	75,461	64.9%	ref.	ref.	71,521	48.5%	ref.	ref.
Higher	23,965	54.3%	0.59	< 0.001	25,524	56.7%	0.60	< 0.001	24,917	41.1%	0.62	< 0.001
**Green Left (GL)**												
Lower	44,692	62.0%	ref.	ref.	47,846	63.2%	ref.	ref.	38,670	45.0%	ref.	ref.
Higher	57,755	58.6%	1.07	0.196	53,139	62.5%	1.11	0.046	57,768	47.6%	1.21	< 0.001
**Party for the Animals (PvdD)**												
Lower	37,964	61.9%	ref.	ref.	40,832	63.7%	ref.	ref.	38,633	46.7%	ref.	ref.
Higher	64,483	59.0%	1.03	0.561	60,153	62.2%	1.02	0.737	57,805	46.5%	1.03	0.588
**50PLUS (50+)**												
Lower	57,554	58.1%	reference	ref.	55,729	61.0%	ref.	ref.	55,484	46.5%	ref.	ref.
Higher	44,893	62.6%	1.31	< 0.001	45,256	65.0%	1.25	< 0.001	40,954	46.7%	1.10	0.052

*Abbreviations: COR* crude odds ratio, *MMR* mumps-measles-rubella, *DT (aP)-IPV* diphtheria-tetanus-pertussis-polio, *NL* the Netherlands, *Ned Antilles/Aruba* the Netherlands Antilles and Aruba, *WC* western countries, *NWC* non-western countries. *km* kilometer, *VVD* People’s Party for Freedom and Democracy, *PvdA* Labor Party, *PVV* Party for Freedom, *FvD* Forum for Democracy, *SP* Socialist Party, *CDA* Christian Democratic Appeal, *D66* Democrats 66, *CU* Christian Union, *SGP* Reformed Political Party, *GL* Green Left, *PvdD* The Party for the Animals, 50 + =50PLUS. For explanatory notes on the political parties we refer to Supplementary material 1

(a) HPV-uptake % = % of total of girls (N) with a completed HPV-vaccination series. Girls invited in 2012 were offered a three-dose series, girls invited in 2014 and 2017 a 2-dose series.

(b) Socioeconomic status classification; low (≤ − 1.0000), low-intermediate (− 0.9999 to 0.0000), high-intermediate (0.0001–0.9999), high (≥1.0000).

(c) Urbanization classification; Very high: > 2500 addresses per km2, high: 1500–2500 addresses per km2, moderately high: 1000–1500 addresses per km2, low: 500–1000 addresses per km2, very low < 500 addresses per km2.

(d) Voting % classification: higher or lower compared to the national mean.

* For this variable/ invitation year, data from the most recent year available was used (See Table 1 for variable details)

**Table 3:** Multilevel multivariable logistic regression analysis of invitation year 2017, model 1.3, (*n* = 96,007; 99.6% of the girls included in model)

Variable	N	HPV-uptake % ^(a)^	Adjusted OR (AOR)	95% CI	p-value
**MMR-vaccination status**					**< 0.001**
Zero vaccinations	2541	6.0%	reference	ref.	ref.
One vaccination	3155	13.3%	2.38	1.96–2.89	< 0.001
Two Vaccinations	90,311	48.9%	14.69	12.44–17.35	< 0.001
**Ethnicity**					**< 0.001**
NL - NL	70,228	49.4%	ref.	ref.	ref.
NL - Turkey	1021	26.1%	0.37	0.32–0.42	< 0.001
Turkey - Turkey	1800	20.5%	0.27	0.24–0.31	< 0.001
NL - Morocco	760	18.7%	0.23	0.19–0.28	< 0.001
Morocco - Morocco	2920	16.5%	0.20	0.18–0.23	< 0.001
NL- Surinam	924	41.5%	0.75	0.65–0.86	< 0.001
Surinam - Surinam	707	45.7%	0.94	0.81–1.10	0.451
NL - Ned Antilles/Aruba	475	35.6%	0.60	0.49–0.73	< 0.001
Ned Antilles/Aruba - Ned Antilles/Aruba	182	35.7%	0.83	0.60–1.15	0.266
NL - other WC	2320	51.0%	1.07	0.98–1.16	0.142
other WC - other WC	704	46.9%	1.17	0.99–1.37	0.065
NL - other NWC	1667	50.0%	1.03	0.93–1.14	0.555
other NWC - other NWC	2188	51.1%	1.25	1.14–1.37	< 0.001
other WC - other NWC	196	35.7%	0.65	0.48–0.88	0.005
Unknown	9915	42.9%	0.91	0.87–0.95	< 0.001
**Socioeconomic status** ^(b)^					**< 0.001**
Low	18,149	36.9%	ref.	ref.	ref.
Low - intermediate	25,467	44.6%	1.21	1.15–1.27	< 0.001
High - intermediate	34,292	48.8%	1.40	1.34–1.47	< 0.001
High	18,099	54.8%	1.68	1.59–1.77	< 0.001
**Road distance**					**< 0.001**
0 km	13,104	45.8%	ref.	ref.	ref.
0–5 km	37,943	46.9%	0.99	0.94–1.03	0.555
5–10 km	28,925	46.7%	0.93	0.89–0.98	0.006
> 10 km	16,035	46.2%	0.90	0.85–0.95	< 0.001
**Urbanization level** ^(c)^					**0.002**
Very high	19,258	45.3%	ref.	ref.	ref.
High	29,441	46.1%	0.84	0.70–0.995	0.043
Moderately high	17,432	47.9%	0.75	0.62–0.90	0.002
Low	21,360	48.3%	0.89	0.74–1.08	0.244
Very low	8616	44.1%	0.86	0.70–1.05	0.131
**Voting % People’s Party for Freedom and Democracy (VVD)**					
Lower	58,531	43.5%	ref.	ref.	ref.
Higher	37,476	51.3%	1.22	1.12–1.33	< 0.001
**Voting % Labor Party (PvdA), Denk (DENK)**					
Lower	34,742	46.8%	ref.	ref.	ref.
Higher	61,265	46.5%	0.94	0.86–1.04	0.209
**Voting % Party for Freedom (PVV), Forum for Democracy (FvD)**					
Lower	52,033	48.1%	ref.	ref.	ref.
Higher	43,974	44.8%	0.90	0.81–0.99	0.029
**Voting % Socialist Party (SP)**					
Lower	54,491	44.3%	ref.	ref.	ref.
Higher	41,516	49.5%	1.39	1.27–1.53	< 0.001
**Voting % Christian Democratic Appeal (CDA)**					
Lower	70,868	46.8%	ref.	ref.	ref.
Higher	25,139	45.9%	0.89	0.80–0.99	0.026
**Voting % Democrats 66 (D66)**					
Lower	37,814	42.6%	ref.	ref.	ref.
Higher	58,193	49.1%	1.17	1.05–1.30	0.003
**Voting % Christian Union (CU), Reformed Political Party (SGP)**					
Lower	71,226	48.5%	ref.	ref.	ref.
Higher	24,781	41.1%	0.81	0.73–0.91	< 0.001
**Voting % Green Left (GL)**					
Lower	38,470	45.0%	ref.	ref.	ref.
Higher	57,537	47.6%	1.15	1.03–1.30	0.015
**Voting % Party for the Animals (PvdD)**					
Lower	38,403	46.7%	ref.	ref.	ref.
Higher	57,604	46.5%	0.82	0.74–0.91	< 0.001
**Voting % 50PLUS (50+)**					
Lower	55,213	46.5%	ref.	ref.	ref.
Higher	40,794	46.6%	0.99	0.90–1.09	0.814

*Abbreviations: OR* odds ratio, *CI* confidence interval, *MMR* mumps-measles-rubella, *NL* the Netherlands, *Ned Antilles/Aruba* the Netherlands Antilles and Aruba, *WC* western countries, *NWC* non-western countries, *km* kilometer, *VVD* People’s Party for Freedom and Democracy, *PvdA* Labor Party, *PVV* Party for Freedom, *FvD* Forum for Democracy, *SP* Socialist Party, *CDA* Christian Democratic Appeal, *D66* Democrats 66, *CU* Christian Union, *SGP* Reformed Political Party, *GL* Green Left, *PvdD* The Party for the Animals, 50 + =50PLUS. For explanatory notes on the political parties we refer to Supplementary material 1

(a) HPV-uptake % = % of total of girls (N) with a completed HPV-vaccination series (2 doses).

(b) Socioeconomic status classification; low (≤ − 1.0000), low-intermediate (− 0.9999 to 0.0000), high-intermediate (0.0001–0.9999), high (≥1.0000).

(c) Urbanization classification; Very high: > 2500 addresses per km2, high: 1500–2500 addresses per km2, moderately high: 1000–1500 addresses per km2, low: 500–100 addresses per km2, very low < 500 addresses per km2

(d) Voting % classification: higher or lower compared to the national mean.

### MMR- and DT (aP)-IPV-vaccination status

As the correlation between MMR-vaccination status and DT (aP)-IPV-vaccination status was > 0.80 in the multicollinearity analysis, only MMR-vaccination status was included in the multilevel multivariable logistic regression models. In the multilevel univariate and multivariable models MMR-vaccinations status was significant and positively associated with HPV-vaccination uptake (Tables 2, 3 and supplementary material 2 – model 1.1, 1.2), indicating that girls who did not have a completed series of MMR-vaccination had a lower HPV-vaccination uptake.

### Ethnicity

Overall, girls with one or two parents born in another country than the Netherlands (both western and non-western countries) had a significantly lower HPV-vaccination uptake compared to girls whose parents both were born in the Netherlands (Tables 2, 3 and supplementary material 2 – model 1.1, 1.2).

In each invitation year girls with one or two parents born in Morocco or Turkey showed a significantly lower HPV-vaccination uptake compared to girls with two parents born in the Netherlands (Table 3 and supplementary material 2 - model 1.1, 1.2).

Considering the high number of girls of whom the country of birth of one or two parents is unknown in 2012 and 2014, compared to less unknown values in 2017, the effect of change over time on ethnicity could not be compared in a multilevel multivariate logistic regression model.

### Socioeconomic status (SES)

Girls who lived in lower SES postal code areas had a statistically significant lower HPV-vaccination uptake than girls who lived in higher SES postal code areas (Tables 2, 3, and supplementary material 2 - model 1.1, 1.2). In each invitation year the odds of having received a completed series of HPV-vaccination was highest among girls who lived in a high SES postal code area compared to girls who lived in a low SES postal code area, followed by girls who lived in a high-intermediate SES postal code area, and subsequently, girls who lived in a low-intermediate SES postal code area (Tables 2, 3 and supplementary material 2 - model 1.1, 1.2).

### Road distance

In 2017, the multilevel univariate logistic regression model indicated no statistical significant difference HPV-vaccination uptake among girls who lived closer or further away from the vaccination location (Table 2). However, the multivariable models showed that girls who lived in a postal code area which was five or more kilometers from the postal code area of the vaccination location, had a very small but statistically significant lower odds of having received a completed series of HPV-vaccinations compared to girls living in the same postal code area as the vaccination location (Table 3 and supplementary material 2 - model 2.3)., This association was not significant in the multilevel multivariable models of 2012 and 2014 (supplementary material 2 – model 1.1, 1.2).

### Urbanization level

In the multilevel univariate logistic regression model no statistically significant association was found between municipal urbanization level and girls’ HPV-vaccination uptake in 2017 (Table 2). In the multivariable logistic regression analysis (Table 3), girls who were invited for HPV-vaccination in 2017 and lived in a municipality with a high or moderately high urbanization level had a statistically significant lower HPV-vaccination uptake compared to girls who lived in a very high urban municipality. The multilevel multivariable logistic regression models of invitation year 2012 and 2014 showed that girls living in low and very low urban municipalities had a statistically significant higher HPV-vaccination uptake than girls living in very high urban municipalities (supplementary material 2 - model 1.1, 1.2). In the multilevel multivariable logistic regression analysis including the interaction variable invitation year*urbanization level, no statistically significant different effect was found for urbanization level between the invitation years 2012 and 2014. However, in invitation year 2017, the effect of urbanization is statistically significant different from invitation year 2012, i.e. the difference in HPV-vaccination uptake between different levels of urbanization becomes smaller (supplementary material 2 – model 2.4).

### Voting proportions of political parties in national elections

The multilevel univariate and multivariable logistic regression analysis of 2017 showed a positive association between HPV-vaccination uptake and municipal voting proportion for People’s Party for Freedom and Democracy (VVD), Socialist Party (SP), Democrats 66 (D66) and Green Left (GL) (Tables 2 and 3). This indicates that girls who lived in a municipality with a higher voting proportion for these parties, compared to the national mean, had a statistically significant higher HPV-vaccination uptake. A negative association was showed between HPV-vaccination uptake and a municipal voting proportion for Party for Freedom and Forum for Democracy (PVV & FvD), Christian Democratic Appeal (CDA) -only in the multivariable model-, Christian Union and Reformed political party (CU & SGP) and Party for the Animals (PvdD) -only in the multivariable model- (Tables 2 and 3). This indicates that girls who lived in a municipality with a higher voting proportion for these parties, compared to the national mean, had a lower HPV-vaccination uptake.

Girls who lived in a municipality with a higher voting proportion for the populist parties with liberal-conservative views PVV & FvD had a significantly lower HPV-vaccination uptake in 2017, yet, in invitation years 2012 and 2014 either a positive or no statistically significant association between HPV-vaccination uptake and PVV & FvD voting proportion was found (Table 3 and supplementary material 2 – model 1.1, 1.2, 2.7). A strong negative association between the HPV-vaccination uptake and the municipal voting proportions for the conservative Christian parties CU & SGP was found for invitation years 2012, 2014 and 2017 (Table 2, Table 3, supplementary material 2 – model 1.2, 2.1,). This effect does not change over the invitation years (model 2.11).

## Discussion

This study was performed to gain insight into the current relationship between social, economic and cultural determinants and the HPV-vaccination uptake of Dutch adolescent girls and whether the influence of these factors changed over time. Results showed that previous willingness to vaccinate (defined as MMR-vaccination status), ethnicity, socioeconomic status of the postal code area, urbanization level of the municipality, road distance to vaccination location and municipal voting proportions in national elections were predictors for the HPV-vaccination uptake. Subgroups with a lower HPV-vaccination uptake in 2017 were in particular girls who have not received a MMR-vaccination (HPV-vaccine uptake 6.0% versus 48.9% when having received two MMR-vaccinations), who have one or two parents born in Morocco or Turkey (HPV-vaccine uptake 16.5–26.1% versus 49.4% when having two parents born in the Netherlands), who live in an area with a lower socioeconomic status (HPV-vaccine uptake 36.9% versus 54.8% when socioeconomic status is high) and higher voting proportions in municipalities for Christian political parties (CU&SGP) (HPV-vaccine uptake 41.1% versus 48.5% when voting proportions for Christian political parties are lower) and populist parties with liberal-conservative views (HPV-vaccine uptake 44.8% versus 48.1% when voting proportions for populist parties with liberal-conservative views are lower). Besides some changes in political preferences of the population (association between HPV-vaccination uptake and higher voting proportions for populist parties with liberal-conservative views changed with an Adjusted OR (AOR) of 0.86 (95% CI: 0.83–0.90) in 2017 versus 2012) and changes in the association between HPV-vaccination uptake and urbanization level (the difference in HPV-vaccination uptake between different levels of urbanization becomes smaller) we found no clear determinants which could possibly explain the decrease in the HPV-vaccination uptake.

Several groups in the Netherlands are known to have objections against vaccination in general. Among the orthodox Protestants, who live geographically clustered in the so-called Dutch Bible Belt, approximately 40% has not received childhood vaccinations [20]. In addition, people with affinity with an anthroposophical or natural lifestyle could also have a lower willingness to vaccinate [21, 22]. In our multilevel multivariable logistic regression analysis, we used MMR-vaccination status to indicate people with a lower willingness to vaccinate in general. As expected, we found a significantly lower HPV-vaccination uptake among girls who had not received MMR-vaccinations in the past.

Regarding ethnicity, highest HPV-vaccination uptake was found among girls with both parents born in the Netherlands. Lowest uptake was in particular observed for girls with one or two parents born in Morocco or Turkey. This was also found in a study following the catch-up campaign in the Netherlands [7]. In a systematic review, belonging to minority racial or ethnic groups was also found as risk factors for low completion of HPV-vaccination series [23]. Parents of ethnic groups could be less proficient with the Dutch language and not responding to the invitation. Differences in culture and/or religion could also explain this association [24, 25].

Girls living in areas with lower SES appeared to have lower HPV-vaccination uptake than girls living in areas with higher SES. This relation between SES and HPV-vaccination uptake was also shown in a previous study in the Netherlands [7]. Underlying characteristics which play a role in SES are education level, having a payed job and the income of the household. Although vaccination was free of charge, a higher education level will help to better understand the usefulness of HPV-vaccination. In contrast, studies from England, Switzerland and the US showed that vaccination rates were lower in high-income families or in families with higher education [26–28]. Differences in healthcare systems and vaccination programs (i.e. school-based) between countries could lead to discrepancies in the association between SES and HPV-vaccination uptake.

In the most recent invitation year, 2017, a road distance to the vaccination location of more than five kilometers showed in the multilevel multivariable logistic regression model a very small but statistically significant association with a lower HPV-vaccination uptake. In contrast, no significant association was found between road distance to vaccination location and HPV-vaccination uptake in 2012 and 2014. Another Dutch study showed that the average road distance was 5.7 km and was comparable between 2014 and 2017 [29]. People may have become more critical about travel distance nowadays. So, decreasing the road distance by expanding the number of vaccination locations, especially in rural areas, might help to increase the HPV-vaccination uptake but the magnitude of the effect is uncertain. In countries who have a school-based vaccination program (such as the UK and Australia), in which no additional traveling is necessary, the HPV-vaccination uptake is in general higher [30].

In 2012 and 2014, girls living in areas with higher urbanization levels had a lower HPV-vaccination uptake than girls living in areas with lower urbanization levels. However, in 2017, this association was not found. The Dutch study performed among girls eligible for the catch-up campaign in 2009 showed that unvaccinated girls lived in more urbanized areas [9]. In contrast, a study from Switzerland, showed that living in a rural municipality was associated with a lower uptake [28].

Regarding voting proportions in national elections, we found a lower HPV-vaccination uptake in girls living in municipalities with a higher voting proportions for the Christian political parties (CU&SGP), compared to the national mean. The association between high political preference for Protestant Christian parties and low HPV-vaccination uptake was shown before in the Netherlands [7]. Apart from the objections to vaccination in general, various Christian groups have objections to HPV-vaccination in particular, because it concerns protection against a sexually transmitted disease [7, 9, 31]. A study in the US showed that adolescents from households with orthodox religious beliefs were almost 14 times less likely to get vaccinated [32]. In Switzerland, protestant religious groups were also associated with a lower uptake [28].

Also in 2017, a higher municipal voting proportion for populist parties with liberal-conservative views was found to be associated with a lower HPV-vaccination uptake. Previous database studies found that voters for Party for Freedom (PVV) and Forum for Democracy (FvD) may have less confidence in the government, media, and social institutions [15, 16]. Also, some of the PVV & FvD voters believe that the government hides information about the health risks of vaccines [16]. State-level voting patterns in the US, which may reflect population-level differences in cultural norms and social values, are also associated with uptake for adolescence vaccination [14].

In birth cohorts 2002 and 2003, i.e. who were vaccinated in 2015 and 2016, a sharp decrease in vaccination uptake was observed [5]. To study which determinants were associated with the decrease in the HPV-vaccination coverage it was investigated whether the influence of the various determinants changed over time. Results showed that the association with urbanization level was less clear in the invitation year 2017, compared with 2012 and 2014. Also, no association between the municipal voting rate for populist parties with liberal-conservative views was found in 2012 and 2014. However, in 2017 a high percentage of voters for populist parties with liberal-conservative views in the municipality was associated with a lower HPV-vaccination uptake. This might be due to the lower confidence in the government, media and social institutions as mentioned before [15, 16]. Besides the changes in political preferences of the population and changes in the association between HPV uptake and urbanization level we found no clear determinants associated with the decrease in the HPV-vaccination uptake. The decrease in HPV-vaccination uptake may be more associated with a general decrease in trust in the vaccine and/or the fear of adverse events. Social media might have played a role in this.

Tailored strategies are critical in reaching groups with suboptimal vaccination uptake [33]. We were able to identify target groups that are currently associated with a lower HPV-vaccination rate in the Netherlands. Customized information and/or consultation might be useful to implement for low educated natives, girls with Moroccan or Turkish parents, girls with a Christian background and neighborhoods with a high proportion of voters for populist parties with liberal-conservative views to increase the HPV-vaccination uptake among these groups. Literature research also shows that reminders (before the vaccination moment), a no-show policy (such as a new invitation if one did not show up after the first invitation), customized information, feedback of the vaccination rate to professionals and making it easier to get the vaccinations, can lead to an increase the HPV-vaccination rate up to 10–20% [34]. Also other studies have been initiated in the Netherlands to reduce the inequalities in HPV vaccination uptake [35, 36].

Besides the strength that individual data was used on vaccination status to determine the HPV-vaccination uptake, this study has also some limitations. Data on social, economic, cultural and political determinants were not collected for the purpose of this study and only available on postal code level or municipality level. Therefore, associations on these aggregation levels represent the group of individuals within a given area and might not directly apply to an individual. For example, it concerns the voting behavior of adults in the municipality, while these girls were not yet allowed to vote themselves. On the other hand, the decision about vaccination is also mostly made by the parents of the girls. Furthermore, for some determinants data was not available for the specific years included in this study. In this case the most recent data was used. Proportions for the political parties in national elections were only available for 2012 and 2017. For road distance, only data was available for 2014 and 2017. Therefore, the results for 2012 and 2014 should be interpreted with caution. Also, we used home addresses obtained in 2018. Girls might have been moved in the years before, but we think that these movements outweigh each other and therefore had a very small effect on the analyses. Besides that, some variables contained a large number of missings. Especially for ethnicity, which counted low numbers for some categories in all cohorts, especially in 2012 and 2014. This limits the comparability of these variables over time. Besides the investigated determinants, there are other determinants that are possibly associated with the HPV-vaccination uptake. For example, school-education or being the oldest girls in the family (i.e. the first who is eligible for HPV-vaccination). Unfortunately, no information on these or other potential determinants was available in the databases.

## Conclusions

In this study we identified current social, economic and cultural determinants that are associated with HPV-vaccination uptake for public health relevance. Customized information and/or consultation should be prepared for identified target groups that are associated with a lower HPV-vaccination rate. We found no clear determinants which explain the decrease in the HPV-vaccination uptake. The vaccination coverage recently increased again in the Netherlands [37], probably fostered by the Meningococcal ACWY vaccination campaign for adolescents. This shows that it is possible to increase the vaccination coverage and protect more girls against cervical cancer. This positive message might help to increase the HPV-vaccination coverage in the Netherlands further.

## Supplementary Information

**Additional file 1.**

**Additional file 2.**

## Data Availability

All data generated or analysed during this study are included in this published article and its supplementary information files.

## Abbreviations

50+: 50PLUS (party that stands up especially for the interests of people aged 50 and over)
95%CI: 95% confidence intervals
AOR: adjusted odds ratios
CBS: Statistics Netherlands
CDA: Christian Democratic Appeal (Christian-inspired party at the center of the political spectrum)
COR: crude odds ratios
CU: Christian Union (Christian party, with progressive positions in the social and ecological field and conservative positions on ethical issues).
D66: Democrats 66 (reformist social-liberal party)
DENK: Denk (movement for migrants and a “tolerant and solidary society”)
FvD: Forum for Democracy (conservative, right-wing populist Eurosceptic political party)
GL: Green party (progressive party which attaches great importance to sustainability)
HPV: human papillomavirus
MHS: Municipal Health Services
MMR: mumps, measles and rubella
NIP: national immunization program
OR: odds ratios
PvdA: Labor Party (progressive, social-democratic party)
PvdD: The Party for the Animals (testimonial party with as main goals animal rights and animal welfare)
PVV: Party for Freedom (populist party with both conservative, liberal “right” and “left” views)
SCP: The Netherlands Institute for Social Research
SGP: Reformed Political Party (conservative Christian (Reformed) party that wants to conduct politics strictly according to Biblical standards).
SP: Socialist Party (socialist, Eurosceptic party which has a strong local, action-oriented basis)
VVD: People’s Party for Freedom and Democracy (right-wing liberal party with more progressive positions in ethical matters).
