# Supplementary material for: Determinants of HPV-vaccination uptake and subgroups with a lower uptake in the Netherlands
Source: BMC Public Health. 2021 Oct 12;21:1848. doi: 10.1186/s12889-021-11897-0 (PMC8513172; doi:10.1186/s12889-021-11897-0)
Supplement: Supplementary file 1 — Additional file 1. [file 12889_2021_11897_MOESM1_ESM.docx]

**Supplementary material 1 by manuscript:**

**Determinants of HPV-vaccination uptake and subgroups with a lower uptake in the Netherlands**

A.C. de Munter*^1,2,5^, T.M. Schurink-van t Klooster*^4^, A. van Lier^4^, R. Akkermans*2,3*, H.E. de Melker*4*, W.L.M. Ruijs*1,4*

**Shared first author*

*^1^ Department of Infectious Disease Control, Public Health Service Gelderland-Zuid, The Netherlands*

*^2^ Radboud University Nijmegen Medical Centre, Department of Primary and Community Care & IQ Health care, Nijmegen, The Netherlands.*

*^3^ Radboud University Medical Centre, Radboud Institute for Health Sciences, IQ healthcare, Nijmegen, The Netherlands.*

*^4^ Centre for Infectious Disease Control, National Institute for Public Health and the Environment, Bilthoven, The Netherlands.*

*^5^ GGD GHOR Nederland, Utrecht, The Netherlands*

Corresponding author:

T.M. Schurink-van ‘t Klooster

Department National Immunization Program

Center of Epidemiology and Surveillance of Infectious Diseases

National Institute for Public Health and the Environment

PO box 1

3720 BA Bilthoven

The Netherlands

Tel: (+31) 30 274 3559

Table S1.1. Political parties included in the in the Dutch House of Representatives

| **Abbreviation in Dutch** | **Party’s name in English** | **Political movement / political spectrum** |
| --- | --- | --- |
| VVD | People's Party for Freedom and Democracy | Right-wing liberal party with more progressive positions in ethical matters |
| PvdA | Labor Party | Progressive, social-democratic party |
| DENK* | Denk | movement for migrants and a "tolerant and solidary society” |
| PVV | Party for Freedom | Populist party with both conservative, liberal "right" and "left" views |
| FvD* | Forum for Democracy | Conservative, right-wing populist Eurosceptic political party |
| SP | Socialist Party | Socialist, Eurosceptic party which has a strong local, action-oriented basis |
| CDA | Christian Democratic Appeal | Christian-inspired party at the center of the political spectrum |
| D66 | Democrats 66 | Reformist social-liberal party |
| CU | Christian Union | Christian party, with progressive positions in the social and ecological field and conservative positions on ethical issues |
| SGP | Reformed Political Party | Conservative Christian (Reformed) party that wants to conduct politics strictly according to Biblical standards |
| GL | Green Left | Progressive party which attaches great importance to sustainability |
| PvdD | The Party for the Animals | Testimonial party with as main goals animal rights and animal welfare |
| 50+ | 50PLUS | Party that stands up especially for the interests of people aged 50 and over |
| * only in the Dutch House of Representatives in 2017 | | |

Figure S1.1
